# Consumption of “Diabetes Risk Reduction Diet” and Odds of Breast Cancer Among Women in a Middle Eastern Country

**DOI:** 10.3389/fnut.2022.744500

**Published:** 2022-04-08

**Authors:** Sara Ebrahimi Mousavi, Amir Bagheri, Sanaz Benisi-Kohansal, Leila Azadbakht, Ahmad Esmaillzadeh

**Affiliations:** ^1^Students' Scientific Research Center, Tehran University of Medical Sciences, Tehran, Iran; ^2^Department of Clinical Nutrition, School of Nutritional Sciences and Dietetics, Tehran University of Medical Sciences, Tehran, Iran; ^3^Department of Community Nutrition, School of Nutritional Sciences and Dietetics, Tehran University of Medical Sciences, Tehran, Iran; ^4^Obesity and Eating Habits Research Center, Endocrinology and Metabolism Molecular-Cellular Sciences Institute, Tehran University of Medical Sciences, Tehran, Iran; ^5^Department of Community Nutrition, School of Nutrition and Food Science, Isfahan University of Medical Sciences, Isfahan, Iran

**Keywords:** diabetes risk reduction diet score, breast cancer, insulin, diabetes, cancer

## Abstract

**Background:**

Given the role of insulin resistance in several cancers, we hypothesized that consumption of a diet that reduces insulin resistance might lower the risk of breast cancer.

**Objective:**

The present study was designed to assess the association between consumption of “diabetes risk reduction diet” (DRRD) and odds of breast cancer among a large group of women in a Middle Eastern country.

**Methods:**

This population-based case-control study enrolled 350 newly diagnosed cases of stage I-IV breast cancer and 700 age-matched apparently healthy individuals as controls. We collected dietary data *via* a validated 106-item Willett-format semi-quantitative dish-based food frequency questionnaire. A DRRD score was included based on 9 dietary factors (cereal fiber, coffee, nuts, whole fruits, ratio of polyunsaturated to saturated, trans fat, sugar-sweetened beverages, red and processed meat, and lower glycemic index). For food and nutrient items with a protective association with diabetes in earlier studies, participants were given the score as the quintile of that food item, but for food groups with unfavorable association with diabetes, we did vice versa. Total DRRD score ranged from 5 to 45.

**Results:**

Mean age of cases and controls was 65.28 and 61.04 years. Mean BMI of patients with breast cancer and controls was 25.5 and 21.0. We found that individuals with the greatest adherence to the DRRD were 0.41 times less likely to have breast cancer than those with the lowest adherence (OR: 0.59; 95% CI: 0.38, 0.90, and *P*-trend = 0.002). Stratified analysis by menopausal status indicated a significant inverse relationship in postmenopausal women (OR: 0.57; 95% CI: 0.36–0.90), but not in premenopausal women (OR: 0.76; 95% CI: 0.19–2.96). Moreover, by BMI status, we found statistically significant inverse association between adherence to the DRRD and odds of breast cancer among normal-weight women (OR: 0.59; 95% CI: 0.36, 0.98) but not in overweight women (OR: 0.66; 95% CI: 0.31, 1.40). **Conclusions:** Significant inverse associations were found between adherence to DRRD and breast cancer, especially among postmenopausal and normal-weight women.

## Introduction

The prevalence of breast cancer and mortality due to cancer is higher in patients with diabetes than in healthy individuals (1, 2). A meta-analysis found that people with diabetes had a 20% higher risk of breast cancer (3). Diabetes is usually characterized by insulin resistance, hyperglycemia, and elevated inflammatory markers that is associated with overexpression and progression of breast cancer through different pathways (4–6). Moreover, Obesity is associated with an increased risk of cancer in women (7, 8). Obesity can directly or indirectly provoke cell growth, anti-apoptotic effects, migration, and angiogenesis by increase the levels of adipokines, androgen sex hormones, chronic inflammation as well as hyperinsulinemia (7, 9–11). Therefore, in addition to traditional strategies for preventing breast cancer, lifestyle modifications targeting the obesity/diabetes link may also help treat and prevent breast cancer.

In terms of dietary factors, construction of a dietary pattern including high glycemic index (12–14), refined grains (15, 16), red meat (17–19), and saturated and trans fatty acids (20, 21) may increase insulin resistance, while fiber (22, 23), polyunsaturated fatty acids (24), legumes (25), nuts (26), and fruits (27) might reduce this complication. These dietary factors are also associated with the risk of breast cancer. Diabetes Risk Reduction Diet (DRRD) is a dietary index consisting of these items that can affect insulin resistance, including sugar-sweetened beverage (SSBs), coffee, nuts, and red and processed meats, in addition to the four components included GI, cereal fiber, ratio of polyunsaturated to saturated fat (P: S), and trans-fat (27). Limited studies have examined the association of DRRD with chronic disease (28, 29). A study by Rhee et al. (29) indicated that a higher DRRD score was associated with a 40% reduction in the risk of diabetes. Another cohort study conducted by Kang et al. in 2020 on 88,739 women found that a higher DRRD score was inversely associated with risk of breast cancer (independent of weight change) (28).

To date, no study has examined DRRD score with risk of breast cancer in the Middle Eastern population, where the risk of diabetes and breast cancer is increasing and the traditional dietary pattern is different from current typical diets in Western countries (30). Consumption of large amounts of carbohydrates, especially in the form of refined grains, greater intake of saturated and trans fatty acids and low consumption of fruits, vegetables, legumes, and polyunsaturated fatty acids in these regions has led to the pattern of unhealthy diets in these countries and a high potential for insulin resistance (31). The aim of current study was therefore to investigate the association between diabetes risk reduction diet and odds of breast cancer.

## Materials and Methods

This case-control study was performed on women over 30 years of age between 2013 and 2015 in Isfahan, Iran. We recruited cases from patients who were referred to private hospitals or clinics or were being treated for breast cancer, including tumor resection, chemotherapy, or radiotherapy, or all of them. Breast cancer was pathologically confirmed in cases who had been diagnosed with this condition in the past 6 months through physical examination and mammographic findings. Given that patients were only asked to answer some of the questionnaires, more than 90% of them accepted to take part in the study. The sample size was calculated considering the type I error as 5% and the study power as 80%. In addition, we hypothesized that unhealthy dietary pattern might increase the odds of breast cancer by 1.5 times. Considering the common ratio of 0.25 and the ratio of controls to cases as 2, we needed ~350 patients with breast cancer and 700 apparently healthy controls. In this study, we did not include people with a history of any neoplastic lesions or cysts (except breast cancer) and those who had previously been treated with any hormone replacement therapy. In addition, we did not include people on a special diet in the project. The control group was consisted of Iranian women who had no history of malignancy, cysts, or pathological disease and had no specific diet or hormone replacement therapy. Healthy women in the control group were matched with cases in terms of age and socioeconomic status. Individuals in the control group were chosen from healthy women with no family history of breast cancer by multi-stage cluster random sampling. Finally, a total of 1,050 eligible women, including 350 cases and 700 controls, were recruited to participate in our study. All participants provided informed written consent. The study was ethically approved by the Bioethics Committee of Tehran University of Medical Sciences, Tehran, Iran.

### Dietary Intake Assessment

We collected usual dietary intakes of subjects using a 106-item Willett-format semiquantitative dish-based food frequency questionnaire (FFQ), which was designed specifically for Iranian adults. Details of developing this questionnaire as well as further information about its validity and reliability were reported elsewhere (32). All the questionnaires were filled in a face-to-face interview by a trained dietitian. The questionnaire consisted of five sets of foods and dishes, including (1) mixed dishes: cooked or canned foods (29 items); (2) carbohydrate-based foods: various types of bread, cakes, biscuits, and potato (10 items); (3) dairy products [dairies, butter, and cream (9 items)]; (4) fruits and vegetables (22 items); and (5) accessory food items and beverages: sweets, fast foods, nuts, desserts and beverages (36 items). We asked participants to report their frequency for each food and mixed food. The frequency response categories were different from “never/ less than once per month” to “12 or more times per day.” We calculated the daily amount of each food ingredient considering the serving size and the average frequency reported. In order to estimate mean energy and nutrient intakes for each study subject, Nutritionist IV software was applied, in which the main database was USDA food composition table which was modified for Iranian local foods using the nutrient composition of Iranian food items.

### Construction of DRRDS

Based on earlier knowledge about the association between various food items and diabetes, DRRDS was constructed using previously published reports. Nine food items including cereal fiber, nuts, caffeine, whole fruits, ratio of polyunsaturated fat to saturated fat, GI, trans fats, SSBs/fruit juices, and red and processed meats were considered in this scoring method. First, we classified participants in terms of quintiles of these components. For food and nutrient items with a protective association with diabetes in earlier studies [cereal fiber, nuts, coffee (caffeinated and decaffeinated), whole fruits (raisins, prunes, bananas, cantaloupes, watermelon, fresh apples/pears, oranges, grapefruits, strawberries, blueberry, and peaches/apricots/plums) and the ratio of polyunsaturated fat to saturated fat], participants were given the score as the quintile of that food item; that is individuals in the highest quintile of these foods and nutrients were given the score of 5 and those in quintiles 1, 2, 3, and 4 were given the scores of 1, 2, 3, and 4, respectively. For nutrients and food groups [GI, trans fats, SSBs/fruit juices (apples, oranges, grapefruits, and other juices), and red and processed meats] with unfavorable association with diabetes, we did vice versa; such that individuals in the highest quintile were given the score of 1 and those in the lowest quintile received the score of 5. Similarly, individuals in quintiles 2, 3, and 4 were given the scores of 4, 3, and 2, respectively. Total DRRDS was computed by summing up the scores of participants received for all nine components. This score ranged from 5 to 45 (28).

### Assessment of Breast Cancer

The diagnosis of breast cancer was done using physical examination and mammography. Pathological evaluation was also performed to confirm the diagnosis. We included women of Iranian nationality who had been diagnosed with breast cancer for the maximum of previous 6 months and also had stage I–IV breast cancer.

### Assessment of Other Variables

Required data on other variables such as socio-demographic characteristics (including age, marital status, residence area, and education), past medical history, family history of breast cancer, history of breastfeeding and dietary supplement use dietary supplements (Calcium, Iron, multivitamins, etc.) were collected *via* pretested questionnaires. In order to assess the level of physical activity, the short form of the International Physical Activity Questionnaire (IPAQ) was used, in which the metabolic equivalents-hour per week (MET-h/week) was computed to state the activity level for each study participant (33). Weight was measured using a digital scale with light clothing. Height was assessed by a wall-mounted tape-meter in standing position without shoes. We measured waist circumference (WC) at the middle of the lower rib margin and iliac crest while people were standing and breathing normally. Finally, body mass index (BMI) was calculated as weight divided by the square of height.

### Statistical Methods

We categorized participants into quartiles according to DRRD adherence. In order to compare general characteristics of study population across quartiles DRRD adherence, one-way ANOVA for continuous variables and Chi-square test for categorical variables were applied. Dietary intakes of study participants across categories DRRD adherence were evaluate by ANOVA, with Tukey's *post-hoc* comparison for pairwise differences. To examine the relationship between DRRD adherence and breast cancer, multivariate logistic regression was used in several models for all participants. The obtained findings were modified for confounding factors including age, residence, marital, menopausal and socioeconomic status, education, family history of Breast cancer, breast feeding, history of disease, and dietary supplement use. Further adjustment for BMI was applied in the last model. Stratified analyses by menopausal status as well as BMI were also conducted. We used quartile categories as an ordinal variable, in order to assess the trend of odds ratios across increasing quartiles of DRRD score. All analyses were done *via* Statistical Package for Social Sciences (SPSS Corp, version 19, Chicago, IL, USA). *P* < 0.05 were identified as statistically significant.

## Results

A total of 1,050 female participants with a mean age of 62.5 years were included in the study. Number (percentage) of individuals with BMI < 25 kg/m^2^ was 271 (77.4%) among cases and 352 (50.2%) among controls. Also, the participants with BMI ≥ 25 kg /m^2^ were 79 (22.5%) in the cases and 348 (49.7%) in the control group. General characteristics of the study population across quartiles of DRRDS are presented in Table 1. Patients in the top quartile of DRRDS were more likely to be younger, premenopausal, alcohol user, and smoker than those in the lowest quartile (*P* < 0.01 for all). Moreover, a lower percentage of them were residing in urban areas. No other significant differences were found across categories of DRRDS in terms of other general characteristics.

**Table 1:** Characteristics of the study participants across subjects with and without breast cancer and also across quartile categories of DRRDS^*^.

	**Quartiles of DRRDS**				***P* ^†^**
	**Q1 (*n* = 292)**	**Q2 (*n* = 233)**	**Q3 (*n* = 283)**	**Q4 (*n* = 242)**	
Age (years)	64.29 ± 10.02	61.95 ± 10.64	62 ± 11.61	61.26 ± 10.48	<0.01
BMI (kg/m^2^)^*^	24.05 ± 5.46	24.17 ± 5.17	24.69 ± 5.48	24.39 ± 4.95	0.49
Physical activity (Met/h)	34.84 ± 6.51	35.58 ± 7.14	34.85 ± 6.59	35.06 ± 6.34	0.57
Urban-resided (%)	42.5	33.5	36	31	0.03
Family history of breast cancer (%)	5.5	5.6	3.9	7	0.47
History of diseases (%)	9.2	6	9.9	11.6	0.20
University graduated (%)	23.3	20.6	28.3	27.7	0.14
Alcohol use (%)	3.4	7.3	9.2	6.2	0.04
Smoker (%)	11.2	14.2	16.6	18.6	0.01
Married (%)	81.2	83.3	84.1	86.8	0.11
Supplement use (%)	7.9	10.7	10.2	11.2	0.57
Post menopause (%)	86.6	80.3	77.7	78.9	0.03
History of breast feeding (%)	36.6	33.5	34.3	30.2	0.47

* *All values are mean ± SD, unless indicated*;

† *ANOVA for continuous variables and Chi-squared test for categorical variables*.

Dietary intakes of the study population across quartiles of DRRDS are displayed in Table 2. There were significant differences between quartile of DRRDS and dietary intakes of energy, carbohydrates, proteins, dietary fiber, cereal fiber, nuts, caffeine, whole fruits, PUFA to SFA ratio, sugar-sweetened beverages, red and processed meats, fats, trans fatty acids, and GI (*P* < 0.001). The Tukey's *post-hoc* analysis was performed to compare pairwise differences between dietary intakes of study participants across quartile categories of DRRDS in Table 3. Accordingly, participants in the third category of DRRDS were significantly higher intakes of energy, carbohydrate, protein, fiber, fiber, grains, nuts, and whole fruits than participants in the first category (*P* < 0.001 for all). In contrast, the consumption of fat (*P*: 0.012), trans fatty acids (*P* < 0.001), and red and processed meats (*P* < 0.049) of participants in the third category was lower than that of the first category. Also, individuals in the top quartile of DRRD had higher energy (*P*: 0.001), carbohydrate (*P* < 0.001), protein (*P* < 0.001), fiber (*P* < 0.001), cereal fiber (*P* < 0.001), nuts (*P*: 0.003), caffeine (*P* < 0.001), whole fruits (*P*: 0.012), higher PUFA to SFA ratios (*P*: 0.010), and GI (*P*: 0.006), than participants in the lowest quartile. In addition, those with the highest quartile had lower intakes of fat (*P* < 0.001), trans fatty acids (*P* < 0.001), SSBs / fruit juices (*P*: 0.002), and red and processed meats (*P* < 0.001), vs. those with the lowest quartile.

**Table 2:** Dietary intakes of study participants across quartile categories of DRRDS^*^.

	**Quartiles of DRRDS**				***P* ^†^**
	**Q1 (*n* = 292)**	**Q2 (*n* = 233)**	**Q3 (*n* = 283)**	**Q4 (*n* = 242)**	
**Nutrients**
Energy (kcal/d)	2,115.88 ± 681.03	2,326.52 ± 744.69	2,373.94 ± 647.33	2,345.00 ± 672.77	<0.001
Carbohydrate (g/d)	265.55 ± 85.15	321.90 ± 115.09	339.72 ± 99.45	348.94 ± 110.94	<0.001
Protein (g/d)	67.89 ± 26	75.67 ± 26.30	82.04 ± 27.51	85.74 ± 31.59	<0.001
Fat (g/d)	91.94 ± 39.99	87.95 ± 37.42	83.0.0711 ± 31.18	74.26 ± 26.73	<0.001
Fiber (g/d)	18.85 ± 6.51	22.11 ± 7.72	24.54 ± 8.13	24.48 ± 7.70	<0.001
**Food items**
Cereal fiber (g/d)	10.79 ± 4.86	13.33 ± 6.15	14.82 ± 5.67	15.93 ± 5.93	<0.001
Nuts (g/d)	1.21 ± 3.90	2.14 ± 4.84	3.25 ± 7.84	2.89 ± 4.96	<0.001
Caffeine (mg/d)	169.74 ± 133.72	282.09 ± 252.30	280.20 ± 195.61	314.85 ± 230.08	<0.001
Whole fruits (g/d)	138.49 ± 162.74	154.02 ± 131.55	188.61 ± 181.83	179.33 ± 120.38	<0.001
PUFA to SFA	0.15 ± 0.07	0.24 ± 0.26	0.29 ± 0.25	0.42 ± 0.38	<0.001
GI	62.37 ± 2.62	62.88 ± 2.41	62.86 ± 2.37	63.06 ± 2.18	0.007
Trans fatty acids (g/d)	0.58 ± 0.38	0.47 ± 0.29	0.38 ± 0.25	0.23 ± 0.16	<0.001
SSBs/fruit juices (g/d)	2.05 ± 4.11	1.49 ± 2.88	1.68 ± 3.59	0.92 ± 3.62	0.04
Red and processed meats (g/d)	14.31 ± 14.96	11.26 ± 12.51	11.02 ± 20.99	6.08 ± 8.63	<0.001

* *All values are mean ± SD*.

† *ANOVA for all variables*.

*PUFA to SFA, Ratio of polyunsaturated fatty acid to saturated fatty acid; GI, Glycemic index; SSB, Sugar-sweetened beverages*.

**Table 3:** Pairwise comparison of the dietary intakes across each quartile categories of DRRDS^*^.

**Variables**	**Q_**1**_, Q_**2**_**	**Q_**1**_, Q_**3**_**	**Q_**1**_, Q_**4**_**	**Q_**2**_, Q_**3**_**	**Q_**2**_, Q_**4**_**	**Q_**3**_, Q_**4**_**
**Energy (kcal/d)**
Mean deference	−210.64	−258.06	−229.12	−47.41	−18.47	28.94
*p*	0.003	<0.001	0.001	0.862	0.991	0.963
**Carbohydrate (g/d)**
Mean deference	−56.35	−74.17	−83.39	−17.82	−27.03	−9.21
*p*	<0.001	<0.001	<0.001	0.200	0.021	0.733
**Protein (g/d)**
Mean deference	−7.78	−14.15	−17.85	−6.37	−10.07	−3.69
*p*	0.008	<0.001	<0.001	0.048	0.001	0.428
**Fat (g/d)**
Mean deference	3.98	8.82	17.67	4.83	13.69	8.85
*p*	0.551	0.012	<0.001	0.384	<0.001	0.018
**Fiber (g/d)**
Mean deference	−3.25	−5.69	−5.62	−2.43	−2.37	0.06
*p*	<0.001	<0.001	<0.001	0.001	0.003	1.00
**Cereal fiber (g/d)**
Mean deference	−2.54	−4.03	−5.14	−1.48	−2.59	−1.10
*p*	<0.001	<0.001	<0.001	0.015	<0.001	0.114
**Nuts (g/d)**
Mean deference	−0.92	−2.03	−1.68	−1.11	−0.75	0.35
*p*	0.239	<0.001	0.003	0.116	0.462	0.889
**Caffeine (mg/d)**
Mean deference	−112.34	−110.45	−145.11	1.89	−32.76	−34.65
*p*	<0.001	<0.001	<0.001	1.00	0.298	0.212
**Whole fruits (g/d)**
Mean deference	−15.52	−50.11	−40.83	−34.59	−25.31	9.28
*p*	0.656	0.001	0.012	0.053	0.274	0.900
**PUFA to SFA**
Mean deference	−3.54	−2.50	−9.06	1.03	−5.52	−6.56
*p*	0.600	0.791	0.010	0.984	0.279	0.118
**GI**
Mean deference	−0.504	−0.48	−0.68	0.02	−0.18	−0.20
*p*	0.082	0.078	0.006	1.00	0.842	0.768
**Trans fatty acids (g/d)**
Mean deference	0.10	0.20	0.34	0.09	0.24	0.14
*p*	<0.001	<0.001	<0.001	0.001	<0.001	<0.001
**SSBs/fruit juices (g/d)**
Mean deference	0.56	0.37	1.13	−0.18	0.57	0.75
*p*	0.286	0.598	0.002	0.936	0.313	0.078
**Red and processed meats (g/d)**
Mean deference	3.05	3.29	8.23	0.23	5.17	4.94
*p*	0.104	0.049	<0.001	0.998	0.001	0.001

* *Data was analyzed by Tukey's post-hoc tests*.

*PUFA to SFA, Ratio of polyunsaturated fatty acid to saturated fatty acid; GI, Glycemic index; SSB, Sugar-sweetened beverages*.

Crude and multivariable-adjusted ORs and 95% confidence intervals (CIs) for breast cancer across categories of DRRDS score are outlined in Table 4. Participants with the highest DRRDS score had significantly lower odds of breast cancer than those with the lowest score (OR: 0.66; 95% CI: 0.46, 0.95). After controlling for potential confounding variables, participants with the highest DRRDS were 41% less likely to have breast cancer compared with those with the lowest score (OR: 0.59; 95% CI: 0.39, 0.87, *P*-trend < 0.001). Further adjustment for BMI did not change the mentioned association (OR: 0.59; 95% CI: 0.38, 0.90, *P*-trend = 0.002). When we did the analysis stratified by menopausal status, we found that postmenopausal women with the highest DRRDS score had 37% lower odds for having breast cancer compared with those with the lowest score (OR: 0.63; 95% CI: 0.43, 0.93, *P*-trend = 0.001). This inverse association remained significant after adjustment for several potential confounding variables (OR: 0.55; 95% CI: 0.36, 0.85, *P*-trend < 0.001), even BMI (OR: 0.57; 95% CI: 0.36, 0.90, *P*-trend = 0.001). However, no significant association was seen between DRRDS and odds of breast cancer in premenopausal women, either before (OR: 1.26; 95% CI: 0.48–3.31) or after adjusting for potential covariates (OR: 0.76; 95% CI: 0.19–2.91).

**Table 4:** Multivariable-adjusted odds ratios (95% CIs) for breast cancer across quartile categories of DRRDS, stratified by menopausal status.

	**Quartiles of DRRDS**				***P*-trend**
	**Q1 (*n* = 292)**	**Q2 (*n* = 233)**	**Q3 (*n* = 283)**	**Q4 (*n* = 242)**	
**Breast cancer**
No. of cases (%)	168 (24)	155 (22.1)	215 (30.7)	162 (23.1)	
No. of controls	124 (35.4)	78 (22.3)	68 (19.4)	80 (22.9)	
Crude	1	0.68 (0.47–0.97)	0.42 (0.30–0.61)	0.66 (0.46–0.95)	0.002
Model 1^†^	1	0.59 (0.40–0.87)	0.34 (0.23–0.51)	0.60 (0.41–0.88)	<0.001
Model 2^‡^	1	0.56 (0.37–0.83)	0.33 (0.22–0.50)	0.59 (0.39–0.87)	<0.001
Model 3^§^	1	0.52 (0.34–0.81)	0.35 (0.21–0.51)	0.59 (0.38–0.90)	0.002
**Premenopausal**
No. of cases	9 (22)	9 (22)	9 (22)	14 (34.1)	
No. of controls	30 (19)	37 (23.4)	54 (34.2)	37 (23.4)	
Crude	1	0.81 (0.28–2.29)	0.55 (0.19–1.55)	1.26 (0.48–3.31)	0.73
Model 1	1	0.56 (0.18–1.72)	0.33 (0.11–1.00)	0.82 (0.29–2.29)	0.70
Model 2	1	0.59 (0.16–2.34)	0.46 (0.13–1.53)	0.99 (0.32–3.08)	0.88
Model 3	1	0.37 (0.06–2.12)	0.21 (0.04–0.96)	0.76 (0.19–2.96)	0.86
**Postmenopausal**
No. of cases	115 (37.2)	69 (22.3)	59 (19.1)	66 (21.4)	
No. of controls	138 (25.5)	118 (21.8)	161 (29.7)	125 (23.1)	
Crude	1	0.70 (0.47–1.03)	0.44 (0.29–0.64)	0.63 (0.43–0.93)	0.001
Model 1	1	0.60 (0.40–0.91)	0.34 (0.22–0.52)	0.57 (0.38–0.87)	<0.001
Model 2	1	0.59 (0.38–0.91)	0.33 (0.21–0.50)	0.55 (0.36–0.85)	<0.001
Model 3	1	0.57 (0.36–0.90)	0.34 (0.22–0.54)	0.57 (0.36–0.90)	0.001

† *Model 1: Adjusted for age and energy intake*.

‡ *Model 2: Further adjusted for education, residency, family history of breast cancer, physical activity, marital status, smoking, alcohol consumption, supplement use, breast-feeding, and menopausal status*.

§ *Model 3: Further adjusted for BMI*.

We repeated the regression analysis according to BMI status (Table 5), in which a statistically significant inverse association was observed between DRRDS and risk of breast cancer among normal-weight people, either before (OR: 0.72; 95% CI: 0.46–1.12; *P*-trend = 0.006) or after controlling for potential confounders (0.59; 95% CI: 0.36–0.98; *P*-trend = 0.006). No significant associations were seen between DRRDS and odds of breast cancer in obese women, comparing top vs. bottom quartiles (OR: 0.68, 95% CI: 0.35–1.32, *P*-trend = 0.08). Adjustment for potential confounders did not affect the findings (OR: 0.66; 95% CI: 0.31–1.40; *P*-trend = 0.13).

**Table 5:** Multivariable-adjusted odds ratios (95% CIs) for breast cancer across quartile categories of DRRDS, stratified by BMI.

	**Quartiles of DRRDS**				***P-*trend**
	**Q1**	**Q2**	**Q3**	**Q4**	
**BMI** **<** **25**
No. of cases (%)	98 (36.2)	57 (21)	55 (20.3)	61 (22.5)	
No. of controls (%)	87 (24.7)	77 (21.9)	113 (32.1)	75 (21.3)	
Crude	1	0.65 (0.42–1.02)	0.43 (0.28–0.66)	0.72 (0.46–1.12)	0.02
Model 1^†^	1	0.55 (0.34–0.90)	0.36 (0.22–0.57)	0.63 (0.39–1.02)	0.009
Model 2^‡^	1	0.53 (0.32–0.89)	0.32 (0.19–0.53)	0.59 (0.36–0.98)	0.006
**BMI** **≥** **25**
No. of cases (%)	26 (32.9)	21 (26.6)	13 (16.5)	19 (24.1)	
No. of controls (%)	81 (23.3)	78 (22.4)	102 (29.3)	87 (25)	
Crude	1	0.83 (0.43–1.61)	0.39 (0.19–0.82)	0.68 (0.35–1.32)	0.08
Model 1	1	0.74 (0.37–1.50)	0.30 (0.14–0.65)	0.61 (0.30–1.24)	0.04
Model 2	1	0.75 (0.35–1.59)	0.35 (0.16–0.80)	0.66 (0.31–1.40)	0.13

† *Model 1: Adjusted for age and energy intake*.

‡ *Model 2: Further adjusted for education, residency, family history of breast cancer, physical activity, marital status, smoking, alcohol consumption, supplement use, breast-feeding, and menopausal status*.

## Discussion

To the best of our knowledge, the current study is the first in the Middle East to investigate the association between DRRDS and odds of breast cancer. In this case-control study among 1,050 Iranian women, we found a significant inverse association between DRRDS and odds of breast cancer. This inverse association was also observed in postmenopausal women, either before or after controlling for confounders. Separately by BMI status, this inverse relationship was seen among normal weight women, but not in overweight or obese people.

Breast cancer is a growing public health concern worldwide (34). Along with rising prevalence of obesity and hyperinsulinemia in the world, prevalence of breast cancer is also increasing dramatically (8). Weight loss and dietary modifications are potentially important strategies to prevent developing insulin resistance and consequently breast cancer (35–37). Following a variety of dietary patterns, including the dietary approaches to stop hypertension (DASH) (38, 39), healthy lifestyle score (HLS) (40), and Mediterranean diets (41), has been reported to reduce the risk of breast cancer, and this association is more commonly found in postmenopausal women and lean individuals.

In this study, we found that DRRDS was associated with a reduced odd of breast cancer. Our results were consistent with previous studies. For example, NHS1 and NHS2 cohort study of 182,654 women found that following the DRRD score reduced their risk of breast cancer by 8% (28). Their study also showed that this inverse relationship was seen especially in postmenopausal women as well as in women with a BMI below 25. Also, Turati et al. (42) indicated that increased adherence to DRRD is associated with a reduced odds of breast cancer in the Italian female population. The results of studies on the association of DRRD adherence with various cancers are also consistent with our results. For example, a study by Esposito et al. (43) found a significant negative relationship between DRRD adherence and endometrial cancer risk. Turati et al. (42) found that consumption of DRRD reduced the risk of pancreatic cancer by 45%. Moreover, another study indicated that greater adherence to DRRD after diagnosis related to improved survival outcomes among a large number of breast cancer survivors (44). The results were also in line with other studies that have reported a lower risk of breast cancer with lower GI in diet (45, 46), lower intake of red and process meat (47), higher fiber intake (48), higher consumption of vegetables and fruits (49), and higher intake of PUFA (50). Therefore, it seems that following a diet to reduce risk of insulin resistance and diabetes might also reduce the risk of breast cancer. A recent systematic review and meta-analysis also found that high intakes of vegetables, fruits, cheese, and soy products and low intakes of red and processed meat was associated with a lower risk of breast cancer (51). Fraser et al. (52) reported that consuming more dairy milk was associated with an increased risk of breast cancer. In another study indicated that higher consumption of fruits and vegetables, and specifically cruciferous and yellow/orange vegetables, may reduce the risk of breast cancer, principally those that are more likely to be aggressive tumors (53). Dydjow-Bendek and Zagozdzon (54) suggested that greater intake of PUFA can decrease the risk of breast cancer, while the low omega-3/omega-6 ratio increases the risk. In terms of physical activity, a study also showed that in women with a family history of breast cancer, physical activity was related with decreased postmenopausal, but not premenopausal, breast cancer risk and was not modified by the extent of family history (55). However, the inverse association was only seen in postmenopausal women, not in premenopausal women in our results. This was also in line with the previous cohort study on this subject (28). This might be attributed to the increased body fat percentage among postmenopausal women, which is associated with increased concentrations of estrogen, insulin, IGF-1 and ultimately with increased mammary gland mass (56, 57). On the other hand, when the ovaries stop producing hormones in postmenopausal women, body fat is the main site of estrogen synthesis, and is also directly associated with hyperinsulinemia in the body (1, 58). Consumption of a DRRD can affect insulin secretion in the long term through which it may protect against the effect of a high estrogenic environment (28). One might assume that small population of premenopausal women in the current study (*n* = 199) may explain lack of a significant relationship between DRRDS and the odds of breast cancer, but it must be kept in mind that such an association was not also seen in a previous cohort study with a large sample size of premenopausal women (28).

An interesting finding of the current analysis was that the protective association between DRRDS and breast cancer was only seen in normal-weight women, but not in overweight and obese participants. The same finding was also reported by investigators in NHS (28). Earlier studies have also shown that consumption of unhealthy diets was strongly associated with elevated risk of T2D in lean women (59). No specific reason is available explaining our findings, but there are some possibilities. It seems that obese women are more likely to have triple-negative breast cancer (TNBC) and normal-weight women are more likely to have human epidermal growth factor receptor (Her2+) breast cancer (60). Human epidermal growth factor receptor 2 is a type of tyrosine kinase growth factor receptor that involves downstream signaling from the PI3K, AKT and mTOR pathways (61–64). Therefore, Her2 is highly associated with hyperinsulinemia pathways and insulin resistance. Earlier studies have also indicated that metformin is inversely related to this subtype of breast cancer, so that metformin can suppress overexpression of Her2 receptors and prevent breast cancer (62, 65, 66).

Insulin and insulin-like growth factor-1 (IGF-1) are the most important negative regulators of the sex hormone-binding globulin (SHBG) synthesis pathway *in vitro* and may lead to the development of breast tumors in a variety of ways (67, 68). In fact, insulin has a high affinity for the IGF-1 receptor, when stimulated, leads to mitogenic and anti-apoptotic pathways in breast cell lines (67, 69). Therefore, diet and lifestyle modifications aimed at reducing insulin resistance can play an important role in reducing the risk of breast cancer. Possible mechanisms by which DRRDS can reduce the risk of breast cancer include reduced hyperinsulinemia, which can in turn lead to decreased levels of hormones involved in the growth and proliferation of mammary gland cells (67, 69). DRRDS can also affect risk of breast cancer by its influence on weight because weight loss by reducing dietary fat can prevent the effects of fat-induced carcinogens. High consumption of cereal fibers, nuts, fruits, vegetables, caffeine, PUFA and less intake of SSB, trans fatty acids and red and processed meat, which are considered in the DRRDS, may contribute to this association (70–73). Thus, DRRDS is rich in antioxidants, dietary fiber, and polyphenols that can mediate the inverse relationship between DRRDS and breast cancer. Thus, DRRDS is rich in antioxidants, dietary fiber, and polyphenols that can mediate the inverse relationship between DRRDS and breast cancer.

The present study contains several strengths. This is the first study in the Middle East to examine the association between DRRDS and breast cancer. Adequate number of studied people, accurate assessment of potential confounders and controlling for them in the analysis are also among the strengths of the present study. Another strength of this study was that the food questionnaires were completed in a face-to-face interview by a trained nutritionist. The use of the USDA food composition table, which was modified for Iranian local foods using the nutrient composition of Iranian food items, was another strength of this study. The limitations of the study include the following points: (1) the study type is case-control which would prevent the inference of causation and (2) this study is subject to selection and recall bias. However, applying the population-based study, we tried to reduce the possibility of selection bias, (3) measurement errors are unavoidable and would lead to misclassification of participants, (4) proportion of postmenopausal women was higher than premenopausal, which might explain finding the expected association in this group only, (5) failure to assess insulin resistance, and (6) lack of data on the hormonal status of breast cancer and medication use in this people are another limitation.

Overall, we found that consumption of “diabetes risk reduction” diet was associated with a reduced odds of breast cancer, in particular among normal-weight women and postmenopausal women. Additional studies in other countries of the Middle East are needed to further examine this hypothesis in the region.

## Data Availability Statement

The raw data supporting the conclusions of this article will be made available by the authors, on reasonable request to the corresponding author.

## Ethics Statement

The studies involving human participants were reviewed and approved by Ethical Committee of Tehran University of Medical Sciences approved the study protocol. The patients/participants provided their written informed consent to participate in this study.

## Author Contributions

LA and SB-K contributed to data collection, assisted in designing the study, conceptualized, and oversaw this study. AB and AE provided substantial contributions to the editing of the paper. SE took primary responsibility for drafting this manuscript. AB and SE provided guidance on the analysis. AE conducted the analysis. All authors contributed to the article and approved the submitted version.

## Funding

The financial support for this study comes from Tehran University of Medical Sciences, Tehran, Iran.

## Conflict of Interest

The authors declare that the research was conducted in the absence of any commercial or financial relationships that could be construed as a potential conflict of interest.

## Publisher's Note

All claims expressed in this article are solely those of the authors and do not necessarily represent those of their affiliated organizations, or those of the publisher, the editors and the reviewers. Any product that may be evaluated in this article, or claim that may be made by its manufacturer, is not guaranteed or endorsed by the publisher.
